# Seasonal dynamics and changing sea level as determinants of the community and trophic structure of oribatid mites in a salt marsh of the Wadden Sea

**DOI:** 10.1371/journal.pone.0207141

**Published:** 2018-11-08

**Authors:** Marlena Winter, Kristin Haynert, Stefan Scheu, Mark Maraun

**Affiliations:** 1 University of Göttingen, J.F. Blumenbach Institute of Zoology and Anthropology, Göttingen, Germany; 2 University of Göttingen, Centre of Biodiversity and sustainable Land Use (CBL), Göttingen, Germany; Fred Hutchinson Cancer Research Center, UNITED STATES

## Abstract

Global change processes affect seasonal dynamics of salt marshes and thereby their plant and animal communities. However, these changes have been little investigated for microarthropod communities. We studied the effect of seasonality and changes in sea level on oribatid mites in the natural salt marsh and on artificial islands in the back-barrier environment of the island Spiekeroog (Wadden Sea, Germany). Three zones of the artificial islands were filled with transplanted sods from the lower salt marsh zone and thereby exposed to three different inundation frequencies. We hypothesized that oribatid mite communities will differ along the natural salt marsh vegetation zones [upper salt marsh (USM), lower salt marsh (LSM), pioneer zone (PZ)], which are influenced by different tidal regimes. Accordingly, total oribatid mite densities declined from the USM and LSM to the PZ. Similarly, oribatid mite species compositions changed along the salt marsh transect and also responded to variations in inundation frequency in LSM on artificial islands with typical species of the USM, LSM and PZ being *Multioppia neglecta* (USM), *Hermannia pulchella* (LSM), *Zachvatkinibates quadrivertex* (LSM, PZ) and *Ameronothrus schneideri* (LSM, PZ). Oribatid mite density in the salt marsh and on the artificial islands was at a maximum in winter and spring; this was due in part to high density of juveniles, pointing to two reproductive periods. We hypothesized that oribatid mite trophic structure changes due to variations in abiotic (e.g., tidal dynamics, temperature) and biotic conditions (e.g., resource availability). Stable isotope (^15^N, ^13^C) and neutral lipid fatty acid analyses indicated that oribatid mite species have different diets with e.g., *Z*. *quadrivertex* feeding on macroalgae and fungi, *A*. *schneideri* feeding on microalgae and bacteria, and *Scheloribates laevigatus* and *M*. *neglecta* feeding on dead organic matter, bacteria and fungi. Overall, the results indicate that oribatid mite species in salt marshes are affected by changes in environmental factors such as inundation intensity, with the effects being most pronounced in species with narrow trophic niches and limited niche plasticity. The results also indicate that oribatid mite communities of the LSM respond little to short-term (one year) changes in inundation frequency.

## Introduction

Salt marshes form the boundary between terrestrial and marine realms and are characterized by harsh and dynamic environmental conditions. They stretch from the mean high-water line to the highest water spring tide levels [1]. Salt marshes provide important ecosystem services associated with plant coverage, such as coastal protection against erosion [2,3].

Climate scenarios predict a continuous sea level rise of about 1–2 m to the end of this century, with accompanying stronger climate extremes [4,5]. This is likely to result in a decline of salt marshes and their associated animal communities, since vegetation may not respond fast enough to cope with rising sea levels and recover from climate extremes [6]. For example, in Königshafen, a tidal bay of the Island of Sylt (northern Wadden Sea, Germany) the mean height of the tide increased by approximately 20 cm from 1930 to 2008, with marked concomitant changes in zonation patterns and species composition of tidal flat animals [7].

Anthropogenic and natural impacts influencing vegetation and macrofauna of salt marshes are well investigated. The transition zone from marine to terrestrial habitats is affected by lunar tides and by seasonality, resulting in strong fluctuations in abiotic and biotic factors. This results in the zonation of the vegetation and affects species compositions of benthic animals [8–10]. Typically, three zones are distinguished, upper salt marsh (USM), lower salt marsh (LSM) and pioneer zone (PZ) [11]. However, little is known on how changing sea levels affect coastal and marine microarthropods, such as oribatid mites (Oribatida), and their seasonal dynamics.

Oribatid mites are mainly soil living animals and, due to their high densities and diversity, are used as model organisms reflecting environmental changes [12,13]. Although most oribatid mites are terrestrial, about 0.5% of known species inhabit salt marsh habitats. Colonization of these habitats is associated with physiological and behavioral adaptations enabling them to cope with fluctuating environmental conditions [8,9], such as salinity. Oribatid mites of the LSM can survive significantly longer submerged in seawater than species of the USM [14] and generally decrease their activity level [15–17]. Some species possess a plastron (a thin external air layer which is linked to tracheae) allowing respiration while inundated [18]. Other species migrate between zones to avoid flooding [19].

Feeding and trophic positions of oribatid mite species were investigated using neutral lipid fatty acid (NLFA) as well as stable nitrogen (^15^N) and carbon (^13^C) isotope analyses. NLFAs are storage products and are catabolized to provide energy for consumers. As lipids in part are incorporated into consumers without change, they provide information on the food consumed [20,21]. Natural variations in stable isotope ratios provide information on the trophic position of consumers and the basal resources used [22–24]. Carbon stable isotope ratios are particularly informative if basal resources include C3 and C4 plants as well as algae as is the case in salt marshes. C3 plants include terrestrial higher plants and some red algae, whereas C4 plants include diatoms, as well as red and green algae [25–27].

Here, we investigate the effect of season on density, community structure and trophic structure of oribatid mites along a salt marsh gradient, and on six vegetated artificial islands in the tidal flats of the island of Spiekeroog in order to study the impact of sea level changes. Three hypotheses were investigated. (1) Total density of oribatid mites differs between vegetation zones along a coastal transect and also in lower salt marsh exposed to different inundation intensity on artificial islands. (2) Total density of oribatid mites fluctuates with season with only a single peak in spring, coinciding with the reproductive period. (3) Trophic structure and nutrition of oribatid mite species differ along salt marsh gradients since the type of food and its availability change along the salt marsh gradient.

## Material and methods

### Study site

The study site was located in the southern part of the island Spiekeroog (53°45’2”- 53°47’1”N, 7°40’0”-7°49’1”E), which belongs to the East Frisian Islands in the Wadden Sea National Park of Lower Saxony. The island is located between Langeoog and Wangerooge and is the fourth largest dune island with an area of 18 km^2^ [28]. Tidal flats and salt marshes prevail in the sheltered southern part of the island facing the mainland [29]. The Wadden Sea National Park of Lower Saxony gave the permission to conduct the study on this site; the study did not involve endangered or protected species.

The zonation of salt marshes is based on vegetation structure driven by inundation frequency depending on shore height [11]. The East Frisian Islands show a characteristic zonation pattern: The USM reaches from 35 cm above the MHWL up to the storm tide limit and is inundated 35–70 times a year and the salinity ranges from 5 to 20 ‰, the flora is dominated by *Elymus athericus*. Characteristic plant species of the LSM are *Atriplex portulacoides* and *Puccinellia maritima*. This zone reaches from 0 to 35 cm above the MHWL and is inundated 150–250 times a year, the salinity ranges between 20 and 26 ‰. Daily inundations prevail in the pioneer zone, which is located below the MHWL, the salinity ranges from 26 to 32 ‰. *Salicornia stricta* and *Spartina anglica* are characteristic inhabitants of this vegetation zone [30].

### Experimental design and sampling

Six plots each of an area of 3.24 m^2^ (1.8 * 1.8 m) were established in each of the three salt marsh zones (USM, LSM, PZ). Each plot was divided into four subplots (0.81 m^2^ each). Two subplots were randomly allocated for non-destructive analyses (i.e., vegetation surveys, seedling counts) and two subplots were allocated for destructive analyses [31]. Two samples were taken in the two destructive subplots, resulting in four samples per plot per vegetation zone (3 vegetation zones x 6 plots x 4 subplots = 72 samples overall). The mean of these four samples was used for statistical analysis to avoid pseudoreplication. Sediment samples were taken with a corer (5 cm Ø) at a depth of 0–5 cm. The initial sampling was performed in September 2014 after reconstruction of the islands, and repeated in March, August, October and December 2015. Animals were extracted by heat [32], transferred into ethanol and identified to species level using Weigmann (2006) [33] and Ermilov et al. (2012 a,b) [34,35]; juveniles and adults were separated.

Similar to the six vegetated plots in the salt marsh, six vegetated artificial islands were placed in the tidal flats of Spiekeroog [31]. The islands allowed simulation of the effect of changing sea levels on plant and soil animal communities in the Wadden Sea. Three height levels each of an area of 3.24 m^2^ were established: 120, 90 and 60 cm above sea level. The heights correspond to the heights of the salt marsh zones studied, i.e. USM, LSM and PZ. At each height, four stainless steel cages (0.81 m^2^ per cage) were placed, resulting in twelve cages per island (for details see [31]). The distance between the artificial islands ranged between 45 and 90 m. The islands were filled with tidal flat sediment up to 10 cm below the top of each cage and planted with sods (approximately 20x20x30 cm) from the LSM on top of the tidal flat sediment. The transplanted LSM sods were excavated from the salt marsh nearby [31]. This allowed analyzing exposure of the LSM animal community to the inundation frequency typical for the three vegetation zones. Exposing all vegetation zones (USM, LSM, PZ) to altered inundation frequencies was not feasible since this would have tripled the number of artificial islands which was beyond our capability. The rationale for focusing on LSM communities was to change inundation frequency for Wadden Sea animals resembling those at PZ and USM and compare the changes to existing communities adapted to these altered inundation conditions, i.e. communities at PZ and USM of the coastal transect. This allowed judgement of the potential and speed of adaptation of LSM communities to environmental changes. The distance of the artificial islands to the pioneer zone plots on Spiekeroog Island was ca. 330 m [31]. Two samples were taken randomly in two of the four cages of each of the heights resulting in four samples per island height per artificial island (3 heights x 6 plots x 4 subplots = 72 samples in total). Similar to the sampling in the salt marsh zones, the mean of the four samples was used for statistical analysis to avoid pseudoreplication.

### Fatty acid analysis

For fatty acid analysis, oribatid mites were sampled from the vegetation zones where they occurred most frequently. The samples were taken at the same time as the specimens for stable isotope analysis: 25 individuals of *Scheloribates laevigatus* (USM), 15 individuals of *H*. *pulchella* (LSM), 25–30 individuals of *Z*. *quadrivertex* (LSM/PZ) and 30 individuals of *A*. *schneideri* (PZ). Since too few individuals of *M*. *neglecta* could be sampled the species was exchanged by *S*. *laevigatus* that represents the USM. The oribatid mites were transferred into snap lid glass vials and stored at -10°C. NLFAs of animals (*Scheloribates laevigatus*, *H*. *pulchella*, *Z*. *quadrivertex*, *A*. *schneideri*) were extracted as described in Haubert et al. (2004) [36]. To separate the fatty acids from other components (proteins and polysaccharides), the lipid fraction was transferred to a silicic acid column and neutral lipids were eluted with chloroform. Then, neutral lipids were dried at ambient temperature using a rotation vacuum concentrator (RVC 2e25, Christ, Osterode, Germany). Lipid fractions were saponified, methylated and washed according the procedure given in the Sherlock Microbial Identification System (MIDI, Newark, USA). Fatty acid methyl esters (FAMEs) were transferred into vials, capped and stored at -21°C until analysis by gas chromatography (Clarus 500, Perkin Elmer, Waltham, USA). The software Total Chrome Version 6.3.2 (2008, PerkinElmer, Waltham, USA) was used to determine the fatty acids due to their retention time and to calculate their proportion in the consumer’s diet.

### Stable isotope analysis

For stable isotope analysis, oribatid mites were sampled from each vegetation zone and artificial island height in September 2014 (initial), March, August, October and December 2015 and extracted by heat. Further, thirteen plant species, six algae species and dead organic material (OM) were collected by hand along the coastal transect and on the artificial islands. The material was dried, weighed and transferred into tin capsules (HEKAtech GmbH, Wegberg, Germany). Twenty individuals of *Multioppia neglecta*, two of *Hermannia pulchella* and *Ameronothrus schneideri* adults and five to six of juveniles, and six individuals of *Zachvatkinibates quadrivertex* were pooled for individual stable isotope measurements. Potential resources including C3 and C4 plants, algae and organic matter were distinguished based on location and stable isotope values [37]. C3 plants are mainly terrestrial higher plants with δ^13^C values ranging from -21 to -35 ‰, whereas δ^13^C values of C4 plants, diatoms and green algae range from -10 to -14 ‰ [25]. OM from the USM and LSM included mainly decaying C3 plants, and OM from the PZ included mainly decaying C4 plants and algae.

Natural variations in stable isotope ratios (^15^N/^14^N and ^13^C/^12^C) were analyzed using an elemental analyzer (Euro EA 3000, EuroVector S.p.A; Milano Italy) modified for measuring small samples and coupled with an isotope mass spectrometer (Delta V Plus, Thermo Electron, Bremen Germany) [38]. Stable isotope ratios were expressed as δX (‰) = [(R_sample_—R_standard_)/R_standard_] x 1000, with X representing the target isotope and R the heavy-to-light isotope ratios (^13^C/^12^C and ^15^N/^14^N) of the sample and standard, respectively. Vienna PD Belemnite (PDB) and atmospheric nitrogen served as primary standard for δ^13^C and δ^15^N, respectively. Acetanilide was used for internal calibration.

### Statistical analysis

Variations in total oribatid mite density with sampling date (September 2014, March, August, October, December 2015) and between the three vegetation zones (USM, LSM, PZ) as well as between the three heights of the artificial island (120, 90, 60 cm) were analyzed using analysis of variance (ANOVA) with the fixed factors “season” and “vegetation zone” respectively “island height”. Means were inspected using Tukey’s honestly significant differences test (HSD). Data were log_10_-transformed prior to the analysis.

The density of the four most abundant oribatid mite species of the coastal transect and the artificial islands (*M*. *neglecta*, *H*. *pulchella*, *Z*. *quadrivertex*, *A*. *schneideri*) was analyzed using two-factorial multivariate analysis of variance (MANOVA, Pillai’s Trace; [37]) with the fixed factors “season” (September 2014, March, August, October, December 2015) and “vegetation zone” (USM, LSM, PZ) (coastal transect data) or “island height” (120, 90, 60 cm) (artificial island data). To identify which of the taxa contributed most to the effects detected by the MANOVA, separate ANOVAs for these taxa were performed (‘protected ANOVAs’, [39]). The density of juvenile and adult *H*. *pulchella* of the three vegetation zones and artificial island heights was analyzed as described above. Differences between means were inspected by Tukey’s HSD test (p<0.05). The analyses were performed in R 3.3.1 (R Development Core Team 2016).

Amounts of neutral lipid fatty acids (NLFAs) of oribatid mite species were converted into percentages and arcsine-square root transformed. NLFA patterns were analyzed using two-factorial multivariate analysis of variance (MANOVA, Pillai’s Trace; [37]) with the fixed factors “oribatid mite species” (*H*. *pulchella*, *Z*. *quadrivertex*, *S*. *laevigatus*, *A*. *schneideri*) and “vegetation zone” (USM, LSM, PZ). For analyzing differences in NLFA proportions in the diet of the four species (*S*. *laevigatus*, *H*. *pulchella*, *Z*. *quadrivertex* and *A*. *schneideri*) subsequent ANOVAs were performed for each NLFA using oribatid mite species as fixed factor. Here, only *S*. *laevigatus* was used as representative species of the USM since the number of *M*. *neglecta* in the samples was too low for fatty acid analysis. Differences between means were inspected using Tukey’s HSD post hoc test. For analyzing differences in fatty acid composition in the diet of oribatid mites of different vegetation zones principal components analysis (PCA) of percentage fatty acid values including all four oribatid mite species were performed using CANOCO 5 (Microcomputer Power, Ithaca, USA, 2012). Oribatid mite species were included as passive variables.

Differences in stable isotope values of oribatid mite species were analyzed separately for each vegetation zone and artificial island height using discriminant function analysis (DFA) with δ^13^C and δ^15^N values as independent variables and species as grouping variable. Further, stable isotope values of potential resources [organic matter, C4 plants and micro- and macroalgae and C3 plants (terrestrial plants) and some algae] were analyzed using ANOVA with the fixed factors “resource type” (organic matter, C3 plant, C4 plant) followed by Tukey’s HSD post hoc test. Statistical analyses were performed using Statistica 7 (StatSoft, Inc.; Tulsa, OK, USA). To estimate the potential food proportion of oribatid mite species, Bayesian mixing models were used based on means of stable isotope signatures in the program Fruits 2.1.1 Beta [40].

## Results

### Total density

**Fig 1:**
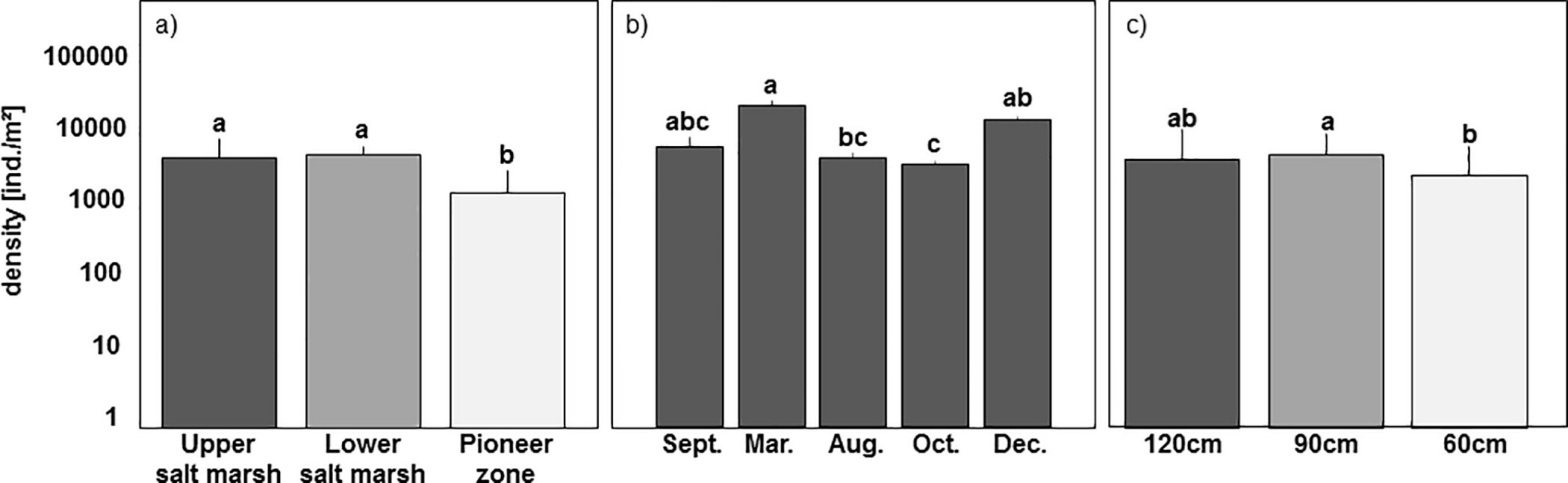
(a) Density (means+SD; note log-scale) of total oribatid mites (adults and juveniles) from the upper salt marsh, lower salt marsh and pioneer zone. (b) Density (means+SD; note log-scale) of total oribatid mites from all three vegetation zones at different sampling dates. (c) Density (means+SD; note log-scale) of total oribatid mites from the three artificial island heights (120, 90, 60 cm).

On the coastal transect total density of oribatid mites (adults and juveniles) differed significantly between the three vegetation zones (F_2,75_ = 9.14, p<0.001; Fig 1A) and the five sampling dates (F_4,75_ = 8.14, p<0.001; Fig 1B). It was similar in the USM and LSM (3529 ± 2634 and 3774 ± 1041 ind./m^2^, respectively) but lower in the PZ (1213 ± 1170 ind./m^2^). Total density was at a maximum in March and December 2015 and at a minimum in August and October 2015.

On artificial islands total density of oribatid mites did not differ significantly between seasons (overall mean 3390 ± 1660 ind./m^2^; F_4,75_ = 2.20, p = 0.077), but differed significantly between the three island heights (F_2,75_ = 4.92, p = 0.010; Fig 1C). It was similar at 120 and 90 cm (3563 ± 5401, 4296 ± 3771 ind./m^2^) but lower at 60 cm (2312 ± 2994 ind./m^2^).

### Density of species

**Fig 2:**
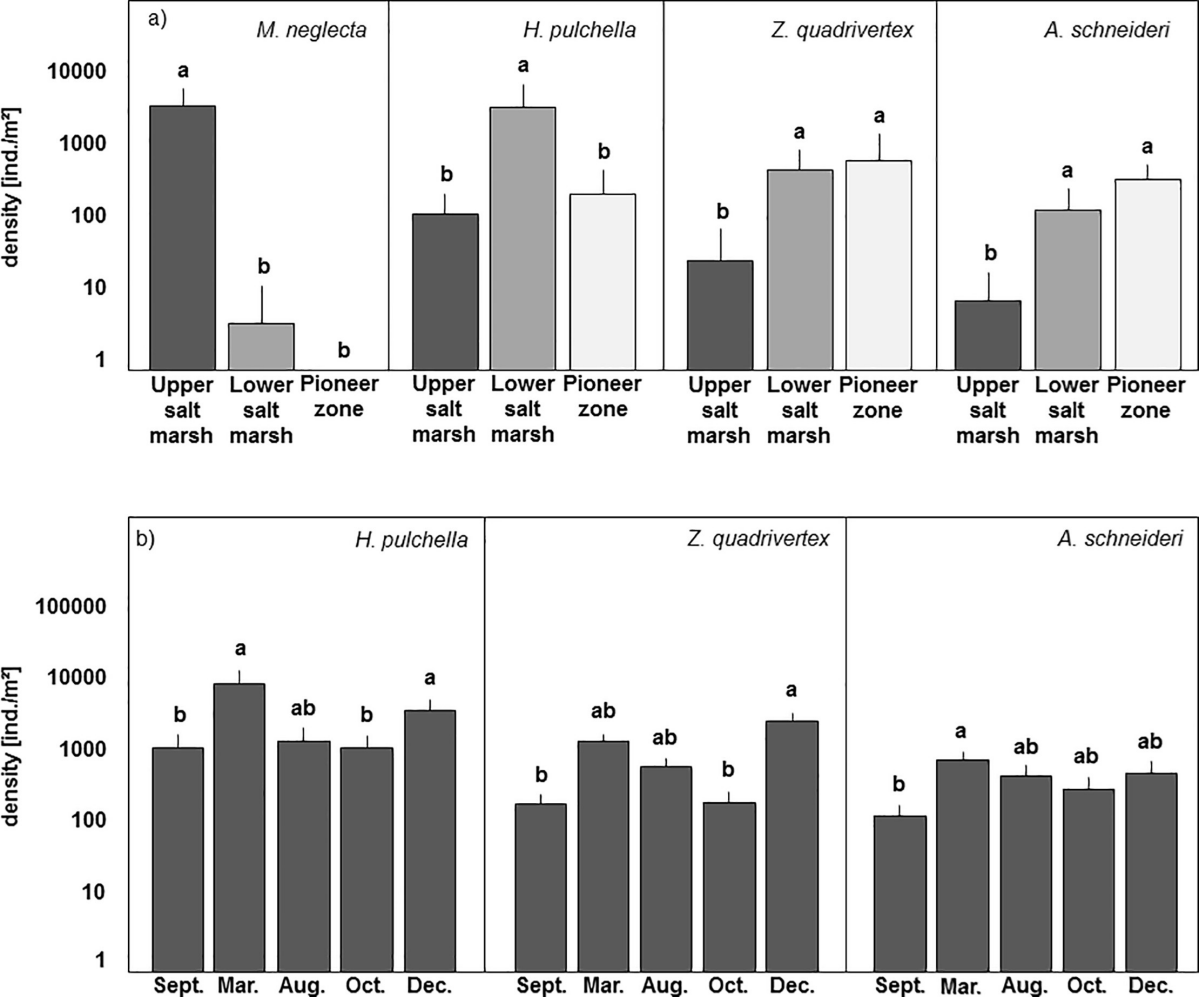
(a) Density (means+SD; note log-scale) of *Multioppia neglecta*, *Hermannia pulchella*, *Zachvatkinibates quadrivertex* and *Ameronothrus schneideri* (contributing to the MANOVA effect) from the three vegetation zones. (b) Density (means+SD; note log-scale) of *Hermannia pulchella*, *Zachvatkinibates quadrivertex* and *Ameronothrus schneideri* (contributing to the MANOVA effect) from the three vegetation zones at different sampling dates. Bars sharing the same letter do not differ significantly (Tukey’s HSD test, p>0.05).

MANOVA indicated that the densities of the four oribatid mite species on the coastal transect (*M*. *neglecta*, *H*. *pulchella*, *Z*. *quadrivertex*, *A*. *schneideri*) varied between vegetation zones (Pillai’s Trace, F_2,75_ = 43.58, p<0.001) and also with season (Pillai’s Trace, F_4,75_ = 2.482, p = 0.0014). There was no significant interaction of vegetation zone and season (Pillai’s Trace, F_8,75_ = 0.457, p = 0.206). Subsequent univariate analysis of variance indicated that the MANOVA effect of vegetation zone was due to all four species: *M*. *neglecta* occurring only in the USM (3368 ± 2897 ind./m^2^), *H*. *pulchella* with its density at LSM (3159 ± 1014 ind./m^2^) exceeding that at USM (123 ± 87 ind./m^2^) and PZ (225 ± 265 ind./m^2^), *Z*. *quadrivertex* with high densities at PZ (636 ± 938 ind./m^2^) and LSM (462 ± 273 ind./m^2^) and low density at USM (30 ± 41 ind./m^2^), and *A*. *schneideri* with its density at PZ (352 ± 63 ind./m^2^) exceeding that at LSM (140 ± 83 ind./m^2^) and at USM (8 ± 13 ind./m^2^) (‘protected ANOVAs’: F_2,75_ = 239.56, p<0.001; F_2,75_ = 45.47, p<0.001; F_2,75_ = 15.93, p<0.001; F_2,75_ = 34.42, p<0.001; for *M*. *neglecta*, *H*. *pulchella*, *Z*. *quadrivertex* and *A*. *schneideri*, respectively; Fig 2A). In contrast, the MANOVA effect of season was only due to three species: *H*. *pulchella* contributed mainly to the effect with high densities in March and December 2015 and low densities in September 2014, August and October 2015 (‘protected ANOVA’: F_4,75_ = 7.53, p<0.001; Fig 2B), and *Z*. *quadrivertex* with high density in December and March 2015, but low density in August and September 2014 as well as October 2015 (‘protected ANOVA’: F_4,75_ = 4.24, p = 0.0034; Fig 2B). *Ameronothrus schneideri* contributed only little to the MANOVA effect of season with highest densities in March and December 2015 (‘protected ANOVA’: F_4,75_ = 2.64, p = 0.04; Fig 2B).

MANOVA further indicated that the densities of juvenile and adult *H*. *pulchella* varied significantly with season (Pillai’s Trace, F_4,75_ = 4.02, p<0.001) and vegetation zone (Pillai’s Trace, F_2,75_ = 19.37, p<0.001). Subsequent ANOVAs indicated that the response of juveniles and adults was similar and therefore the data were pooled (see above, Fig 2A and 2B). Overall, juveniles contributed 58.5% to total density of *H*. *pulchella* at the coastal transect.

**Fig 3:**
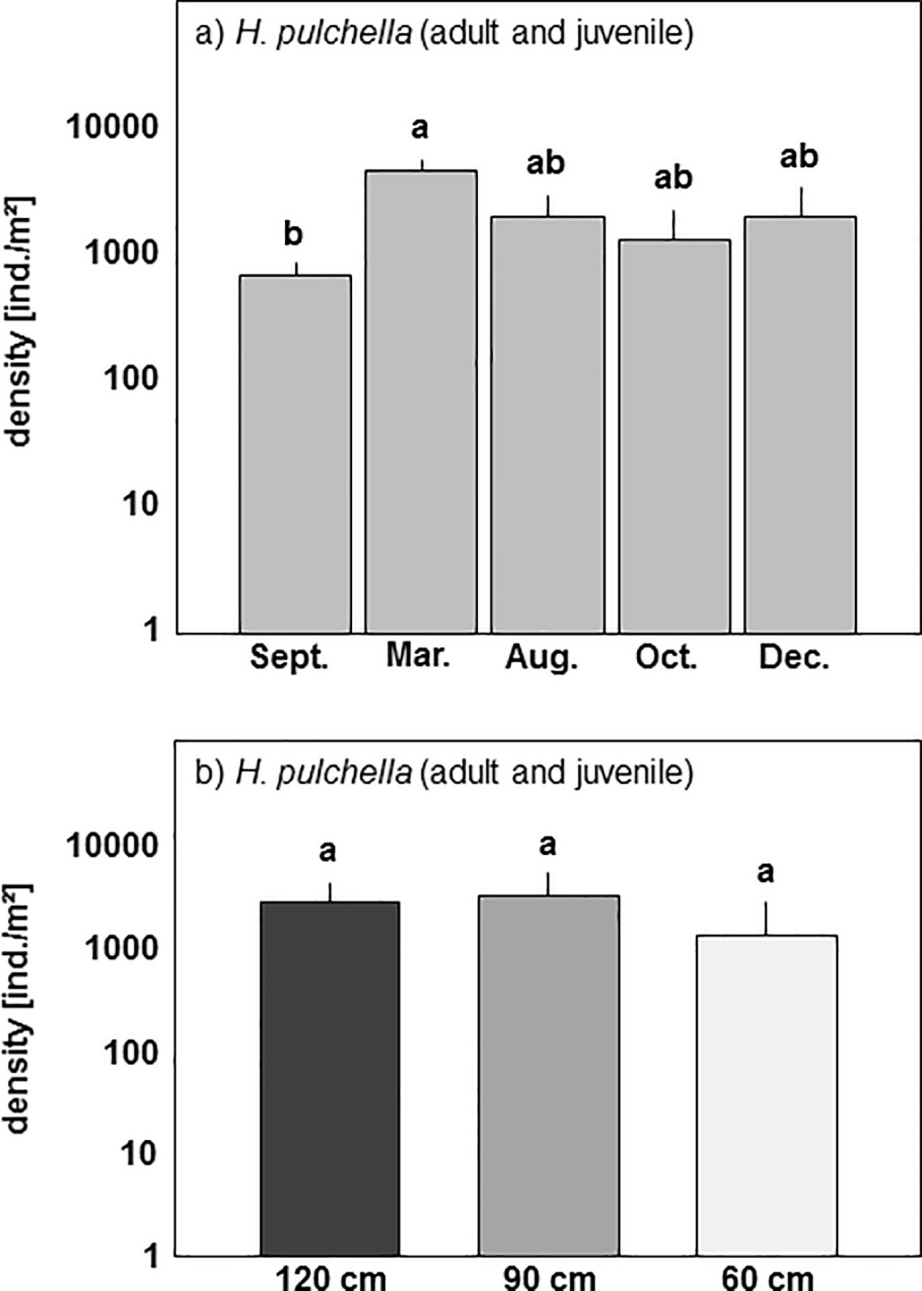
(a) Density (means+SD; note log-scale) of *Hermannia pulchella* (adults and juveniles) on the artificial islands at different sampling dates (Sept., September 2014; March, March 2015; Aug., August 2015; Oct., October 2015; Dec., December 2015) and (b) density (means+SD; note log-scale) of *Hermannia pulchella* (adults and juveniles) from three artificial island heights (120, 90, 60 cm). Bars sharing the same letter do not differ significantly (Tukey’s HSD test, p>0.05).

Similar to the coastal transect, densities of the three oribatid mite species of the artificial islands (*H*. *pulchella*, *Z*. *quadrivertex*, *A*. *schneideri*) varied significantly with season (MANOVA: Pillai’s Trace, F_4,75_ = 1.99, p = 0.026) but also with island height (Pillai’s Trace, F_2,75_ = 2.28, p = 0.039). Subsequent ANOVAs indicated that the significant MANOVA effect was mainly due to *H*. *pulchella* (pooled juveniles and adults). Its density was higher in March 2015 than at the other sampling dates (‘protected ANOVA’: F_4,75_ = 2.53, p = 0.048; Fig 3A). Also, the density of *H*. *pulchella* varied with island height ‘protected ANOVA’: F_2,75_ = 5.21, p = 0.0076; Fig 3B). Its density at 120 and 90 cm (2798 ± 1870, 2265 ± 1334 ind./m^2^, respectively) exceeded that at 60 cm (1182 ± 1133 ind./m^2^); however, the means did not differ significantly (Tukey’s HSD test, p>0.05; Fig 3B). Overall, juveniles contributed 45.5% to total density of *H*. *pulchella* on the artificial islands.

### Fatty acids

**Fig 4:**
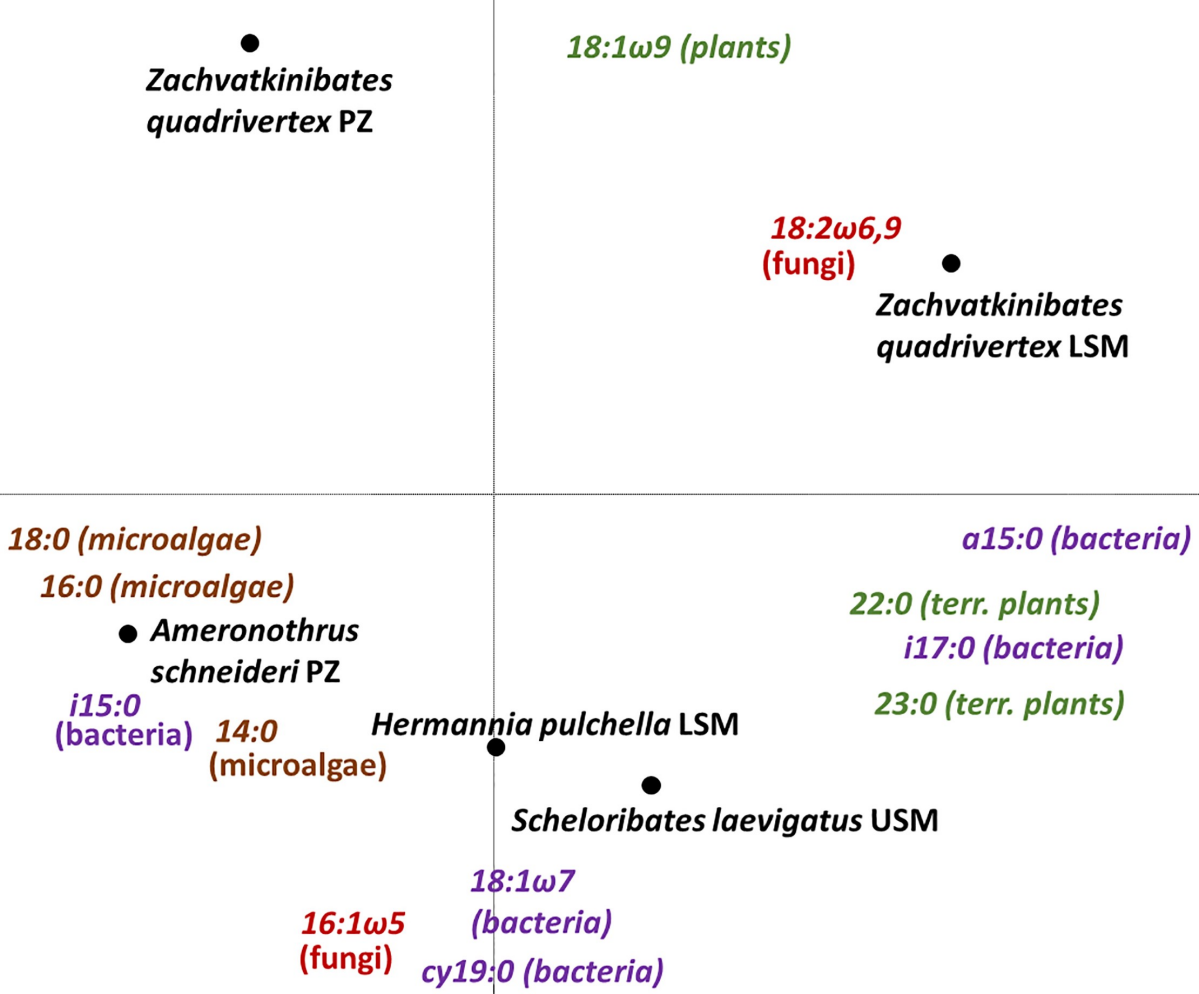
Principal components analysis (PCA) of oribatid mites (*Scheloribates laevigatus*, *Hermannia pulchella*, *Zachvatkinibates quadrivertex*, *Ameronothrus schneideri*) from three salt marsh zones [upper salt marsh (USM), lower salt marsh (LSM), pioneer zone (PZ)] and the body tissue fatty acids 18:1ω7, 18:1ω9t, i15:0, i17:0, cy19:0, 16:1ω5, 18:2ω6,9, 18:1ω9, 18:3ω3, 18:2ω6t, 22:0, 23:0, 14:0, 16:0 and 18:0. Eigenvalues of axes 1 and 2 of 0.66 and 0.23, respectively (length of gradient: 1.3). Different colors indicate different fatty acid biomarkers: bacterial markers (purple), fungal markers (red), plant markers (green), microalgal markers (brown).

The most abundant fatty acids in the four oribatid mite species were the plant marker fatty acid 18:1ω9, the general fungal marker 18:2ω6,9 and the microalgal marker 18:0 (Table 1). Principal components analysis (PCA) suggested that the fatty acid composition of *Z*. *quadrivertex* differed from each of the other species (Fig 4). The species was positioned close to the plant marker fatty acid 18:1ω9 and the fungal marker 18:2ω6,9. *A*. *schneideri* clustered with the microalgal markers 14:0, 16:0 and 18:0, and the bacterial marker i15:0. *S*. *laevigatus* (USM) and *H*. *pulchella* (LSM) clustered together with the microalgal (14:0), bacterial (a15:0, i17:0, 18:1ω7, cy19:0), AM fungal (16:1ω5) and plant marker fatty acids (22:0, 23:0).

**Table 1:** Characteristic fatty acid biomarkers in salt marsh Oribatida based on literature.

Fatty acid type	FA-biomarker	Food source	References
Saturated	14:00	diatoms, microalgae	[39–41]
Saturated	16:00	microalgae	[39–41]
Saturated	18:00	microalgae	[40,41]
Saturated:	i15:0	gram-positive bacteria	[42,43]
iso methyl-branched			
Saturated:	a15:0	gram-positive bacteria	[42,43]
anteiso methyl-branched			
Saturated:	i17:0	gram-positive bacteria	[42,43]
iso methyl-branched			
Saturated:	cy19:0	gram-negative bacteria	[42,43]
cyclopropyl ring			
Saturated:	22:00	terrestrial plants	[43,44]
> C20			
Saturated:	23:00	terrestrial plants	[43,44]
> C20			
Monounsaturated:	18:1ω7	bacteria	[43]
double bond C7			
Monounsaturated:	16:1ω5	AM fungi	[45,46]
double bond C5			
Monounsaturated:	18:1ω9	plants, fungi, macroalgae, cyanobacteria	[47–49,44]
double bond C9			
Polyunsaturated:	18:2ω6,9	fungi, plants	[50–52,43]
double bond C6, C9			

MANOVA indicated that the NLFA patterns differed between the three vegetation zones (Pillai’s Trace, F_10,12_ = 7.92, p<0.007) and also between the oribatid mite species (Pillai’s Trace, F_15,14_ = 2.46, p<0.049). Overall, species from the USM and LSM contained each of the fatty acids. In contrast, a15:0, i17:0, 22:0 and 23:0 were not found in species from the PZ (S1 Fig). Inspection of the individual fatty acid composition using ANOVAs indicated that three fatty acid markers differed significantly between oribatid mite species. The proportion of each the bacterial marker i15:0 (F_4,8_ = 13.54, p = 0.001) as well as the microalgae markers 14:0 (F_4,8_ = 4.53, p = 0.033) and 18:0 (F_4,8_ = 5.53, p = 0.019) were at a maximum in *A*. *schneideri* (from PZ). In contrast, the proportion of i15:0 and 14:0 were at a minimum in *Z*. *quadrivertex* (from PZ), whereas that of 14:0 was at a minimum in *S*. *laevigatus* (from USM) and *Z*. *quadrivertex* (from LSM).

### Stable isotopes—Coastal transect

#### Basal resources

**Fig 5:**
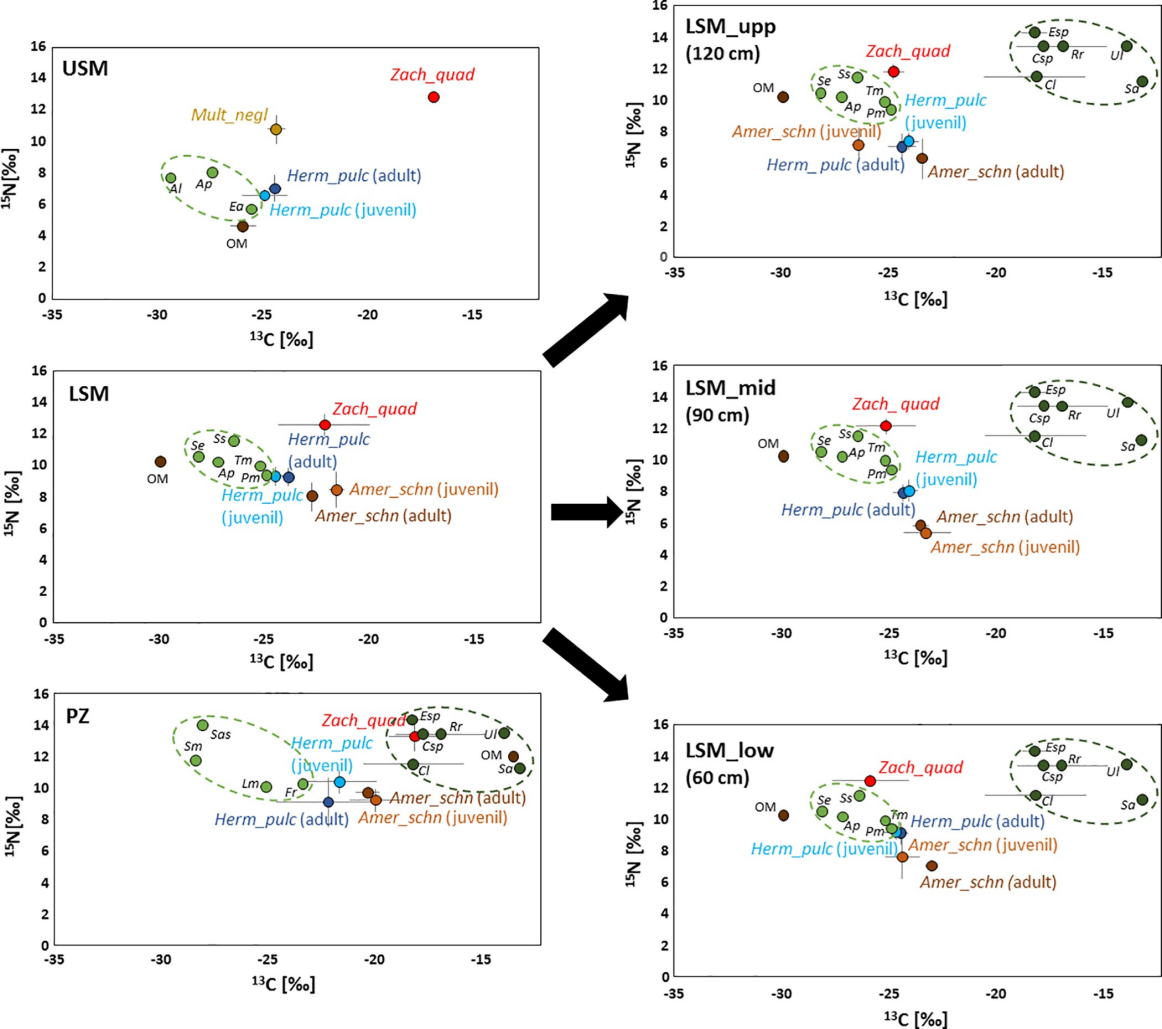
Stable isotope values (δ^13^C [‰] and δ^15^N [‰]) (means ± SD) of *Multioppia neglecta* (Mult_negl; yellow), *Hermannia pulchella* (Herm_pulc; blue) (adults and juveniles), *Zachvatkinibates quadrivertex* (Zach_quad; red), *Ameronothrus schneideri* (Amer_schn; brown) (adults and juveniles) at different salt marsh zones [upper salt marsh (USM), lower salt marsh (LSM), pioneer zone (PZ)] and artificial island heights [LSM_upp (120 cm), LSM_mid (90 cm), LSM_low (60 cm)]. Dotted ellipses in light green (drawn by eye) mark C3 plants (Al- *Atriplex littoralis*, Ap- *Atriplex prostrata* and *Atriplex portulacoides*, Ea- *Elymus athericus*, Pm- *Puccinellia maritima*, Se- *Salicornia europaea*, Ss- *Spergularia salina*, Tm- *Triglochin maritima*, Fv- *Fucus vesiculosus*, Lv- *Limonium vulgare*, Sas- *Salicornia stricta* and Sm- *Suaeda maritima*) and in dark green C4 plants and algae (Csp- *Ceramium sp*., Cl- *Chaetomorpha linum*, Esp- *Enteromorpha sp*., Rr- *Rhizoclonium riparium*, Sa- *Spartina anglica* and Ul- *Ulva lactuca*); org. mat., organic matter (dark brown). Black arrows from the LSM to the three artificial island heights indicate the vegetation zone where soil, plants and species originated from.

δ^13^C and δ^15^N values differed significantly between C3 and C4 plants/algae (grouped according to their δ^13^C values, [37,25]), but both varied markedly (from -29.31 to -23.34 ‰ and -18.21 to -13.17 ‰ in C3 and C4 plants/algae for ^13^C, respectively; F_1,16_ = 109.90, p<0.001; from 5.61 to 13.92 ‰ and 11.19 to 14.30 ‰ in C3 and C4 plants/algae for ^15^N, respectively; F_1,16_ = 10.11, p = 0.006). Further, δ^13^C and δ^15^N values of organic matter differed significantly between the vegetation zones (-25.9 ‰, -29.9 ‰ and -13.45 ‰ in the USM, LSM and PZ for ^13^C, respectively; F_2,3_ = 317.20, p<0.001; 4.59 ‰, 10.23 ‰ and 11.94 ‰ in the USM, LSM and PZ for ^15^N, respectively; F_2,3_ = 223.30, p<0.001; Fig 5).

#### Oribatid mite species

Generally, δ^13^C and δ^15^N values of adult and juvenile *H*. *pulchella* and *A*. *schneideri* varied little (Fig 5) and therefore were pooled for further statistical analyses. In the USM δ^13^C and δ^15^N values of *H*. *pulchella* were on average -24.62 and 6.77 ‰, respectively. In particular, δ^15^N values of *M*. *neglecta* exceeded those of *H*. *pulchella* (DFA: F_2,7_ = 38.09, p<0.001; Fig 5), whereas δ^13^C values of *M*. *neglecta* were only slightly higher than those of *H*. *pulchella*.

In the LSM δ^13^C and δ^15^N values of *H*. *pulchella* where on average -24.15 and 9.27 ‰, respectively. δ^13^C values of *H*. *pulchella* were lower than those of *A*. *schneideri* and *Z*. *quadrivertex* (DFA; F_2,16_ = 15.65, p<0.001), whereas δ^15^N values of *H*. *pulchella* were lower than those of *Z*. *quadrivertex* but higher than those of *A*. *schneideri* (F_2,16_ = 22.89, p<0.001; Fig 5). Additionally, δ^15^N values of *Z*. *quadrivertex* exceeded those of *A*. *schneideri* (DFA; F_2,16_ = 46.67, p<0.001), whereas their δ^13^C values differed little.

In the PZ δ^13^C and δ^15^N values of *H*. *pulchella* and of *A*. *schneideri* were on average -21.93 and 9.65 ‰, and -20.10 and 9.42 ‰, respectively. δ^13^C and δ^15^N values of *Z*. *quadrivertex* significantly exceeded those of both *H*. *pulchella* and *A*. *schneideri* (DFA; F_2,15_ = 10.79, p = 0.001 and F_2,15_ = 13.42, p<0.001, respectively; Fig 5). Additionally, δ^15^N values of *H*. *pulchella* exceeded those of *A*. *schneideri*, whereas δ^13^C values of *H*. *pulchella* were lower than those of *A*. *schneideri* (DFA; F_2,15_ = 3.75, p = 0.048).

#### Resource use of oribatid mites

Mixing models suggested that in the USM *H*. *pulchella* fed mainly on OM (70%) followed by C3 plants (24%). *Z*. *quadrivertex* also fed mainly on OM (64%), followed by C4 plants/algae (21%) and C3 plants (15%). *M*. *neglecta* fed mainly on C3 plants (41%) and on OM (40%). In the LSM *Z*. *quadrivertex* fed on C3 and C4 plants/algae as well as OM in decreasing proportions (39, 35 and 26%). *H*. *pulchella* fed mainly on C3 plants (73%) followed by C4 plants/algae (16%) and OM (11%). *A*. *schneideri* fed mainly on C3 (68%) and less on C4 plants/algae (24%). In the PZ *Z*. *quadrivertex* fed to a similar extent on C3 (31%) and C4 plants/algae (32%) as well as on OM (37%). *H*. *pulchella* fed mainly on C3 plants (66%), followed by C4 plants/algae (18%) and OM (16%). *A*. *schneideri* fed mainly on C3 plants (63%), followed by C4 plants/algae (17%) and OM (20%).

### Stable isotopes—Artificial islands

#### Basal resources

δ^13^C and δ^15^N values differed significantly between C3 plants, C4 plants/algae and OM (from -24.84 to -28.08 ‰, from -18.21 to -13.17 ‰ and -29.9 ‰ in C3 and C4 plants/algae and OM for ^13^C, respectively; F_2,9_ = 47.57, p<0.001; from 9.34 to 11.45 ‰, from 11.19 to 14.30 ‰ and 10.23 ‰ in C3 and C4 plants/algae and OM for ^15^N, respectively; F_2,9_ = 9.20, p = 0.007), but not between the island heights (Fig 5).

#### Oribatid mite species

Generally, δ^13^C and δ^15^N values of adult and juvenile *H*. *pulchella* and *A*. *schneideri* differed little and therefore were pooled for further analyses. In Fig 5, similarities in δ^13^C and δ^15^N values of juveniles and adults of both species are depicted, indicating little variation in resource use. At 120 cm δ^13^C and δ^15^N values of *H*. *pulchella* were on average -24.21 and 7.23 ‰, respectively, and those of *A*. *schneideri* -24.60 and 6.62 ‰, respectively (Fig 5). δ^15^N values of *Z*. *quadrivertex* were higher than those of *A*. *schneideri* and *H*. *pulchella* (DFA: F_2,16_ = 51.48, p<0.001), whereas δ^13^C values of *Z*. *quadrivertex* were higher than those of *A*. *schneideri* but similar to those of *H*. *pulchella* (DFA: F_2,16_ = 50.62, p<0.001).

At 90 cm δ^13^C and δ^15^N values of *H*. *pulchella* were on average -24.18 and 7.98 ‰, respectively. δ^13^C and δ^15^N values of *A*. *schneideri* were on average -23.33 and 5.55 ‰, respectively. δ^15^N values of *Z*. *quadrivertex* were higher than those of *A*. *schneideri* and *H*. *pulchella* (DFA: F_2,16_ = 166.97, p<0.001), whereas δ^13^C values of *Z*. *quadrivertex* were lower than those of *H*. *pulchella* and *A*. *schneideri* (DFA: F_2,16_ = 88.34, p<0.001; Fig 5). Additionally, δ^15^N values of *H*. *pulchella* were higher than those of *A*. *schneideri*, whereas the δ^13^C values were slightly lower (DFA: F_2,16_ = 30.59, p<0.001).

At 60 cm δ^13^C and δ^15^N values of *H*. *pulchella* were on average -24.55 and 9.14 ‰, respectively. δ^13^C and δ^15^N values of *A*. *schneideri* were on average -23.69 and 6.53 ‰, respectively. δ^15^N values of *Z*. *quadrivertex* exceeded those of *A*. *schneideri* and *H*. *pulchella* (DFA: F_2,16_ = 70.36, p<0.001),whereas δ^13^C values of *Z*. *quadrivertex* were lower than those of *H*. *pulchella* and *A*. *schneideri* (DFA: F_2,16_ = 25.32, p<0.001). Additionally, δ^15^N values of *H*. *pulchella* were higher than those of *A*. *schneideri*, whereas the δ^13^C values were lower than those of adult *A*. *schneideri* (DFA: F_2,16_ = 22.60, p<0.001).

## Discussion

### Oribatid mite distribution

Total density of oribatid mites along the salt marsh transect declined from the USM and LSM to PZ confirming our first hypothesis. This indicates that either the amount or quality of resources declines from the USM and LSM to PZ and / or that abiotic conditions deteriorate from higher to lower salt marsh zones. Generally, densities were low (~1200–3800 ind./m^2^) compared to those in soils of temperate, base-rich (~20,000–60,000 ind./m^2^) or boreal forests (~50,000–400,000 ind./m^2^) [13,53]. However, they are similar to densities in resource-poor habitats such as soils of tropical montane rain forests (~2000–10,000 ind./m^2^) [54], indicating that resources (or access to them during the flood-free periods) are one of the major drivers limiting oribatid mite densities.

On the artificial islands, density of oribatid mites was significantly lower at 60 cm than at 90 and 120 cm height. Since all the populations originated from the LSM, the initial composition was the same on each artificial island height with the species adapted to the conditions of the LSM being equivalent to 90 cm height. Lower density at 60 cm might indicate that oribatid mite communities of the LSM are sensitive to fluctuating sea levels and suffer more from frequent inundations (60 cm) than from lower humidity (120 cm), at least over a period of one year. Of the three species present, *H*. *pulchella* generally dominated, resembling the situation in the LSM of the Spiekeroog transect.

In the future, dominance structure of oribatid mite species may shift if the forecast of increasing sea levels [55] holds true. A one year study does not allow long-term predictions, but one aspect of oribatid mite reproduction–the mode of parity–can add insight into possible results of tidal changes. It has been suggested [55] that tidal dynamics limit the occurrence of oviparous (egg-laying) species such as *H*. *pulchella* and *Z*. *quadrivertex*, which require protected sites for egg deposition in the periods between flooding [14]. Larviparous (live-bearing) species such as *A*. *schneideri*, probably would have an advantage since larvae develop protected from tidal disturbances inside the mothers’ body [56]. Females of Larviparous species can survive inundation by floating on water and/or by possessing a plastron. Furthermore, rising sea levels may affect salt marsh vegetation [6] especially since anthropogenic land-use limits the movement of plants to higher elevation. Any such change in tidal plant community is likely to affect the habitat and resources of oribatid mites and thereby oribatid mite community structure.

### Season

Densities of oribatid mites changed with seasons (September 2014—December 2015) confirming our second hypothesis. In contrast to our expectations, however, *H*. *pulchella* and *Z*. *quadrivertex* had two density peaks in spring (March) and winter (December) whereas *A*. *schneideri* only had one, in March 2015. In *H*. *pulchella* densities of juveniles and adults responded in a similar way to seasonality. The patterns suggest that *H*. *pulchella* and *Z*. *quadrivertex* reproduce twice but *A*. *schneideri* only once per year. A similar seasonal pattern occurs in diatoms producing blooms in spring and winter [57,58]. Possibly, there is a link between marine resource availability and seasonal fluctuations in the density of oribatid mites. This is supported by the presence of high amounts of microalgal fatty acid markers in oribatid mites, i.e. NLFAs 14:0, 16:0, 18:0. Presumably, oribatid mite species in salt marshes consume diatoms to a considerable extent. Similar density peaks of *A*. *schneideri*, *Z*. *quadrivertex* and *H*. *pulchella* were observed in June 1963 and in case of the latter species in February 1964 in South Wales [8], though in his study peaks occurred about two months later in the year. This may indicate ongoing climatic change; during the past 50 years spring temperatures increased and reproductive cycle of plants and animals have started earlier in the year [59,60].

Seasonal changes in oribatid mite densities on the artificial islands resembled those at the coastal transect, despite exposure to more extreme conditions, i.e. less frequent inundations in the highest zone (120 cm) and more inundations in the lowest zone (60 cm). The similar density peaks during the study period from September 2014 to December 2015 suggest that the reproductive cycle of oribatid mite species is little affected by inundation frequency. However, as mentioned above total density of *H*. *pulchella* was lower at 60 than at 90 and 120 cm height, indicating that this species suffered from frequent inundation. Overall, our results suggest that frequent inundation limits the density of oribatid mite species and this likely prevails across seasons. However, the reproduction cycle was similar in each of the salt marsh zones suggesting that the optimum season for reproduction is independent from inundation frequency.

### Trophic niches

Trophic niches of the studied oribatid mite species differed among salt marsh zones, confirming our third hypothesis. This indicates that resource availability is closely linked to salt marsh zonation as suggested earlier [8–10]. Presumably, allochthonous material, brought in by the sea, such as marine algae and marine dead organic matter, is mainly available in lower salt marsh zones, whereas autochthonous material, such as terrestrial plants and bacteria colonizing their residues, is only available in higher salt marsh zones. Supporting this conclusion and earlier observations [60–62], fatty acid analysis suggested that salt marsh oribatid mites consume a wide range of resources including bacteria, fungi, microalgae (diatoms), macroalgae and terrestrial plant material. However, fatty acid analysis further suggested that the dietary composition of oribatid mite species in the PZ differed from that in the LSM and USM. In the PZ the diet of oribatid mites lacked terrestrial plant and bacterial markers, whereas in the LSM and USM virtually all marker FAs were present, suggesting either that food consumption varies with tidal height or that the respective food resource was lacking in the PZ. Stable isotope analyses support these suggestions.

Previous laboratory experiments support our finding that salt marsh oribatid mites consume a wide range of resources. E.g. Luxton (1966) [56] found *H*. *pulchella* to feed on bacteria and fungi as well as on decaying wood, and his results also support our finding that *A*. *schneideri* mainly feeds on microalgae and bacteria. He found *Z*. *quadrivertex* to feed mainly on fungi, but, as indicated by FA analysis in our study, this only applied to the PZ, whereas in the LSM its diet mainly consisted of algae. This either may have been due to limited availability of other resources in the PZ or to differences in food consumption. Notably, our results suggest that juvenile oribatid mites feed on the same resources as adults, contrasting in part previous studies showing that the diet may shift during development [56,61].

In contrast to the coastal transect, the trophic position of oribatid mite species on the artificial islands did not differ with island height and inundation frequency. This indicates that differences in resource availability did not significantly affect resource use. However, as indicated by stable isotope analyses *Z*. *quadrivertex*, which preferred C4 plants/algae at the pioneer zone of the coastal transects (diatoms as well as green algae), mainly consumed C3 plants on the artificial islands (mainly terrestrial plants; [63]). Presumably, this was due to the presence of LSM vegetation on each of the island heights.

## Conclusions

Generally, the distribution of oribatid mite species followed the zonation of the salt marsh as defined by plants, e.g. *M*. *neglecta* occurred only in the USM and *H*. *pulchella* mainly occurred in the LSM. Differential colonization of salt marsh zones was accompanied by changes in trophic niches of oribatid mite species. As indicated by fatty acid and stable isotope analyses, species of the USM and LSM (*S*. *laevigatus*, *M*. *neglecta*, *H*. *pulchella*, *Z*. *quadrivertex*) mainly consumed autochthonous terrestrial resources (residues of terrestrial plants and potentially also of algae with associated bacteria and fungi), whereas species from the PZ (*A*. *schneideri*, *Z*. *quadrivertex*) mainly consumed marine allochthonous resources (diatoms, green and brown algae, organic matter with associated fungi). Our results point to trophic plasticity in oribatid mite species in salt marsh habitats. Overall, experimental manipulation of sea water levels on the artificial islands affected oribatid mites of the LSM; the dominance structure was altered, but species composition was little affected.

## Supporting information

S1 Fig Relative contribution (% of total) of neutral lipid fatty acids (NLFAs) (mean + SD) of the four oribatid mite species: *Scheloribates laevigatus* (blue), *Hermannia pulchella* (green), *Zachvatkinibates quadrivertex* (LSM: grey; PZ: red) and *Ameronothrus schneideri* (yellow) in the upper salt marsh (USM), lower salt marsh (LSM) and pioneer zone (PZ). Different letters indicate significant differences (Tukey’s HSD test, p<0.05). Different biomarker fatty acids are grouped together (bacterial, microalgal, fungal, plant and terrestrial plant marker fatty acids).
(TIF)
