# Supplementary figures and images for: Seasonal dynamics and changing sea level as determinants of the community and trophic structure of oribatid mites in a salt marsh of the Wadden Sea
Source: PLoS One. 2018 Nov 8;13(11):e0207141. doi: 10.1371/journal.pone.0207141 (PMC6224107; doi:10.1371/journal.pone.0207141)

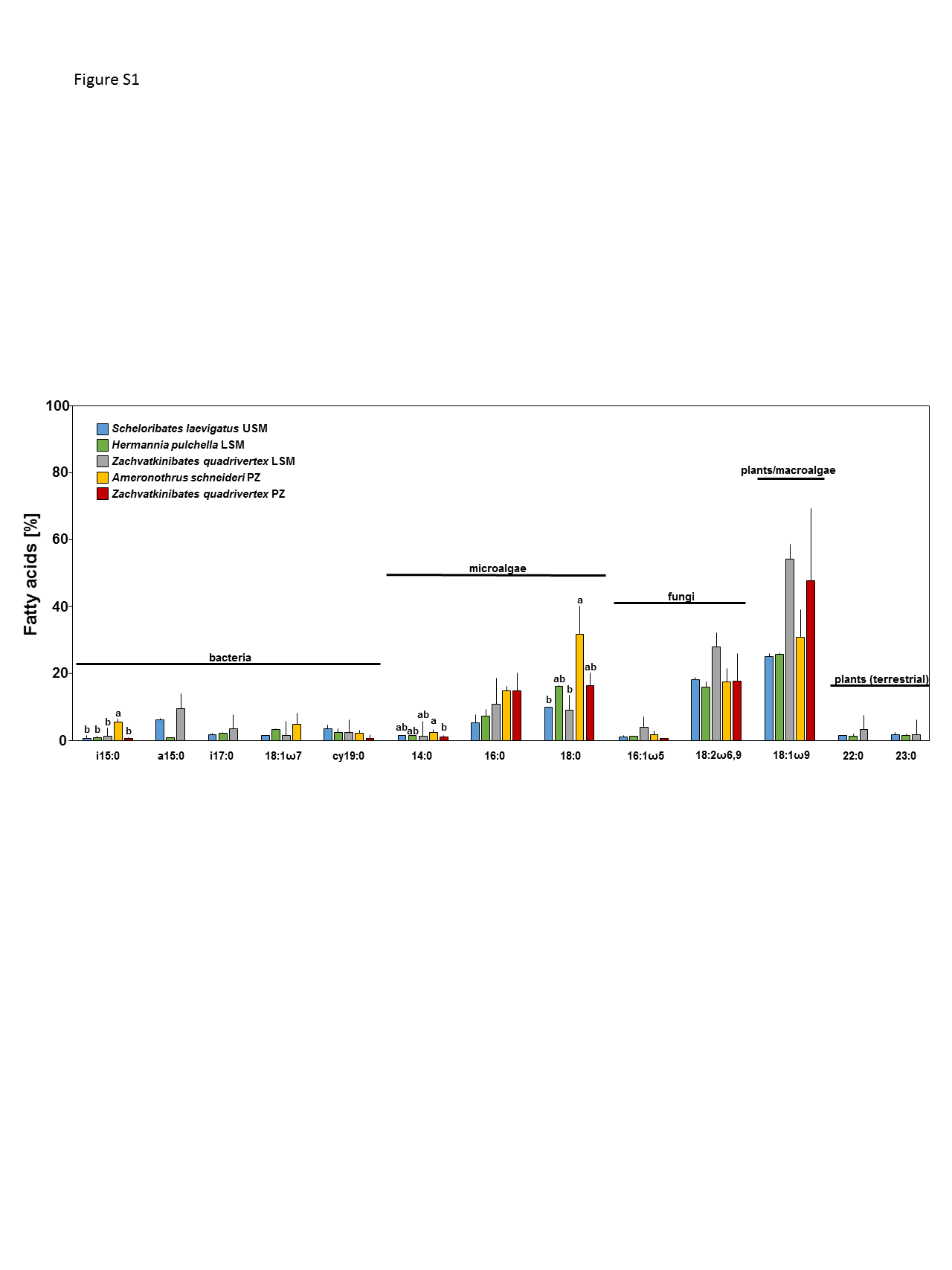

Supplement: S1 Fig — Different letters indicate significant differences (Tukey’s HSD test, p<0.05). Different biomarker fatty acids are grouped together (bacterial, microalgal, fungal, plant and terrestrial plant marker fatty acids). (TIF) [file pone.0207141.s001.tif]
